# From Structure to Behavior in Basolateral Amygdala-Hippocampus Circuits

**DOI:** 10.3389/fncir.2017.00086

**Published:** 2017-10-31

**Authors:** Ying Yang, Jian-Zhi Wang

**Affiliations:** Department of Pathophysiology, School of Basic Medicine and the Collaborative Innovation Center for Brain Science, Key Laboratory of Ministry of Education of China for Neurological Disorders, Tongji Medical College, Huazhong University of Science and Technology, Wuhan, China

**Keywords:** emotion, memory, amygdala, hippocampus, neural circuits

## Abstract

Emotion influences various cognitive processes, including learning and memory. The amygdala is specialized for input and processing of emotion, while the hippocampus is essential for declarative or episodic memory. During emotional reactions, these two brain regions interact to translate the emotion into particular outcomes. Here, we briefly introduce the anatomy and functions of amygdala and hippocampus, and then present behavioral, electrophysiological, optogenetic and biochemical evidence from recent studies to illustrate how amygdala and hippocampus work synergistically to form long-term memory. With recent technological advances, the causal investigations of specific neural circuit between amygdala and hippocampus will help us understand the brain mechanisms of emotion-regulated memories and improve clinical treatment of emotion-associated memory disorders in patients.

## Introduction

Over the past half century, it is increasingly recognized that memories are governed by distinct and interacting brain regions. Medial temporal lobe systems, such as amygdala and hippocampus, have been primarily investigated in emotion associated-memory. The amygdala is specialized for the processing of emotion, while the hippocampus is essential for episodic memory. Thus, the communication between amygdala and hippocampus may serve as a cardinal neural substrates to modify recollection of events at will (Phelps, 2004).

Amygdala and hippocampus can operate independently and interact in subtle ways. Understanding the intricacies of their anatomical structure and their projection circuitry is of great importance given that amygdala and hippocampus are implicated in a wide range of emotional diseases and emotion-associated memory impairment, including anxiety, depression and Alzheimer’s disease (AD), etc. This review will focus on the recent advances that have been promoted by the technologies primarily applied in rodents. Readers are directed to recent reviews for in-depth information on the circuitry of basolateral amygdala and the hippocampus, by which they act synergistically to form long-term memories.

## Anatomy and Physiology of Basolateral Amygdala Subregions

**Figure 1:**
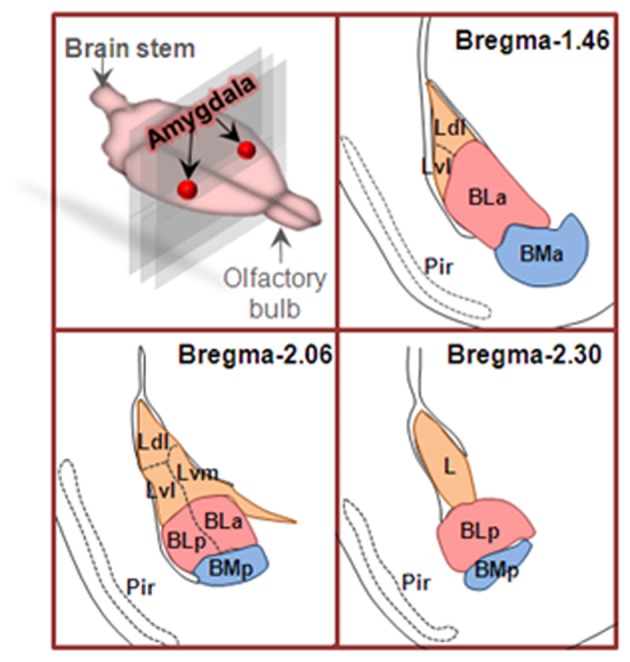
Coronal sections of basolateral amygdaloid complex from rostral to caudal of the brain. Basolateral amygdala groups are divided into three subregions as described in text. Area in orange is lateral nucleus (L), area in pink is basolateral nucleus (BL), and the area in blue is basomedial nucleus (BM). Ldl, dorsolateral part of lateral nucleus; Lvl, ventrolateral part of lateral nucleus; Lvm, ventromedial part of lateral nucleus; BLa, anterior part of basolateral nucleus; BLp, posterior part of basolateral nucleus; BMa, anterior part of basomedial nucleus; BMp, posterior part of basomedial nucleus; Pir, piriform cortex.

Amygdala nuclei are divided into three groups: (1) basolateral amygdala groups (BLA), which contains the lateral nucleus (L or LA), the basal nucleus (BA) and basomedial (BM) nucleus; (2) cortical-like groups, which comprises nucleus of the lateral olfactory tract and the cortical nuclei; and (3) the centromedial groups, which includes the medial and central nuclei (Sah et al., 2003). In the coronal sections from rostral to caudal of the brain, the basal nucleus (also termed basolateral nucleus) can be further divided into arterial part (BLA) and posterior part (BLP). Therefore, BLA has been used to represent basolateral amygdala (Felix-Ortiz et al., 2013) and the anterior part of basolateral nucleus of amygdala (Yang et al., 2016), respectively. To clarify this, the anterior and posterior parts of basolateral nucleus of amygdala are also spelled as BLa and BLp, respectively (Figure 1).

### Lateral Nucleus

Anatomically, the lateral nucleus (LA) is located in the dorsolateral part of the amygdala. It intensively receives extrinsic sensory inputs, meanwhile, sends projections to other amygdala nuclei. So, LA is functionally viewed as an input region of amygdala and origin of many intra-amygdaloidal projections. On the basis of cytoarchitectonics, LA can be further divided into three subdivisions: dorsolateral (the smaller), ventrolateral (the larger) and the medial subdivisions. By means of the anterograde tracer phaseolus vulgaris leucoagglutinin (PHA-L) in LA, intranuclear and internuclear connections have been clearly outlined. The dorsal portion of LA projects to medial and ventral portions, then, the ventral portion in turn projects to the medial division where information will be amply processed within the nucleus (Pitkänen et al., 1995). Study on extranuclear projection showed that LA produces prominent projection to BM nucleus. LA also sends projections to basolateral nucleus, periamygdaloid cortex, the dorsal portion of the central division of the medial nucleus, the posterior cortical nucleus, the capsular division of the central nucleus, and the lateral division of the amygdalo-hippocampal area, but in relatively less magnitude (Pitkänen et al., 1995).

LA is an essential component of amygdala underling fear conditioning memory. In fear learning phases, microinjection of D,L-2-amino-5-phosphovalerate (APV), a broad spectrum of N-methyl-D-aspartate receptor (NMDAR) antagonists into the LA and its adjacent regions, significantly suppresses the acquisition of fear conditioning (Maren et al., 1996; Bauer et al., 2002). Furthermore, selectively blocking GluN2B (NMDAR subunits) by ifenprodil significantly disrupts fear learning without affecting the consolidation of fear memories (Rodrigues et al., 2001). Ca^2+^/calmodulin-dependent protein kinase II (CaMKII) is one of the important downstream effectors of intracellular Ca^2+^ rise via the NMDAR. After fear conditioning, CaMKII undergoes autophosphorylation, then transform to its active form in LA spines. Pharmacological inhibition of CaMKII in LA significantly prevents acquisition but leaving expression of fear memory intact (Rodrigues et al., 2004). Ca^2+^ influx during fear learning also activates protein kinase. Infusion of cyclic AMP-dependent protein kinase (PKA) inhibitor after fear training specifically impairs memory consolidation processes and has no effect on sensory or performance processes (Schafe and LeDoux, 2000). Arc/Arg3.1 protein in the LA is significantly increased after retrieval of an auditory fear memory. Knockdown of Arc/Arg3.1 in the LA impairs fear memory reconsolidation of both a recent and a well-consolidated fear memory (Maddox and Schafe, 2011). Furthermore, local amygdala GABAergic interneurons strongly modulate neural firing in LA and may gate fear learning and/or memory consolidation (Stork et al., 2002; Szinyei et al., 2007; Bergado-Acosta et al., 2008; Johansen et al., 2011), indicating its contribution to fear-related disorders. Therefore, unique molecular and cellular mechanisms in LA may contribute to different stages of fear memory formation.

### Basolateral Nucleus

Basolateral nucleus (BL) is also called basal nucleus. It locates ventrally to the LA and includes three subdivisions, i.e., rostral magnocellular subdivision, caudal intermediate and parvicellular subdivisions. The last two are densely innervated by LA.

BL plays an integral role in anxiety. Patients suffering from anxiety show abnormal activity of BL (Etkin et al., 2009). Concordant with human data, non-specific activation of all glutamatergic BL somata in animals elicits anxiogenic effect (Tye et al., 2011). However, the anxiogenic effect can be abolished or shifted to an anxiolytic effect by stimulating BL terminals in the central nucleus of amygdala (CeL). This finding indicates that majority of BL neurons projecting to downstream targets except CeL mediates ananxiogenic phenotype.

Amygdala volume is positively correlated with social network size and the complexity (Bickart et al., 2011). A recent study shows that inactivation of BL by microinjection of muscimol increases social behavior, while activation of BL by bicuculline significantly suppresses social behavior (Wellman et al., 2016). These findings indicate that BL is a subregion of amygdala that negatively regulates social behavior.

It is also well-established that BL plays a crucial role in reward behavior. BL lesion significantly impairs reward behaviors (Cador et al., 1989; Everitt et al., 1989; Hatfield et al., 1996), while activation of BL-Nac inputs drives reward seeking (Ambroggi et al., 2008; Stuber et al., 2011; Britt et al., 2012; Beyeler et al., 2016). Using mixed appetitive and aversive learning paradigm, Lee et al. (2016) uncovered that the nature of BL neurons activity is to encode behavioral output not encode conditioned stimulation (CS) identity.

Taken together, there is a diversity of neuronal responses in the BL. Precise dissection on BL circuits is essential in anxiety, social and reward studies of BL. It is also required in determining the identity of these unique neurons and elucidating their anatomical connections within BL.

### Basomedial Nucleus

Basomedial nucleus (BM) lies ventrally to the BL and is subdivided into the parvicellular subdivision, the magnocellular subdivision and the intermediate subdivision.

BM bridges the connection between LA and the central nucleus (CeM), which is the output region contributing most amygdala projections to the brainstem fear effectors. Also, BM projects to several anxiolytics brain regions, such as anterodorsal nucleus of the bed nucleus of the striaterminalis (BNST) and the ventral medial prefrontal cortex (vmPFC; Petrovich et al., 1996; Adhikari et al., 2015). But, it does not directly projects to anxiogenic regions, such as the BNST oval nucleus and the dorsal medial prefrontal cortex (dmPFC; Petrovich et al., 1996; Kim et al., 2013). Photoactivation of BM suppresses high-anxiety states and fear-related freezing (Adhikari et al., 2015), while optogenetic or pharmacological inhibition of the BM increases anxiety and freezing (Amano et al., 2011; Adhikari et al., 2015).

## Anatomy and Physiology of Hippocampus Formation

Hippocampus is a critical structure involved in spatial (Buzsáki and Moser, 2013; Geva-Sagiv et al., 2015) and nonspatial memory (Goosens, 2011; Felix-Ortiz and Tye, 2014). Along its longitudinal axis, hippocampus can be functionally divided into dorsal, intermediate and ventral parts (Bannerman et al., 2004; Fenton et al., 2010), and it can be further divided into CA1, CA3 and dentate gyrus (DG) along the transverse axis. There is a canonical trisynaptic loop within hippocampus: from the input node of DG to CA3 and finally to the output node CA1 (Treves and Rolls, 1994; Knierim and Neunuebel, 2016).

CA1 forms the major output of the hippocampus. By classic tracing methods, it has been identified that dorsal CA1 (dCA1) projects to subiculum and entorhinal cortex (Cenquizca and Swanson, 2007), while ventral CA1 (vCA1) targets medial prefrontal cortex (mPFC), nucleus accumbens (NAc) and amygdala (Phillipson and Griffiths, 1985; Jay and Witter, 1991; Friedman et al., 2002; Kishi et al., 2006). Through distinct efferent projections, hippocampus routes selectively behavior-contingent information to the distinct downstreams.

The ventral hippocampus is responsible for affective and motivated behaviors through its distinct target areas. By recording from vCA1 neurons in rats during different behavioral tasks and determining axonal projections with optogenetics, Ciocchi et al. (2015) found that vCA1–prefrontal cortex inputs activates in anxiety-related behaviors; vCA1–NAc inputs activates in goal-directed tasks; and triple-projecting neurons in vCA1, targeting the prefrontal cortex, amygdala, and NAc, are most active during tasks and sharp wave/ripples. In addition, vCA1–NAc shell input is identified and proved to be necessary and sufficient in social memory (Okuyama et al., 2016). It had been presumed that the ventral hippocampus had weak effects on spatial memory due to its large fields and low spatial selectivity. However, Yang et al. (2016) have recently found that stimulating BLP–vCA1 monosynaptic excitatory circuit promotes spatial memory while inhibiting the circuit impairs spatial memory in rats and mice. vCA1’s role in spatial memory is also supported by the evidence that direct vCA1-prefrontal inputs encode spatial cues in spatial working memory (Spellman et al., 2015). Therefore, functional diversity of neurons endows vCA1 with multifarious behavioral phenotypes.

Unlike the ventral part, the dCA1 is primarily associated with spatial navigation and episodic memory. The pyramidal neurons in dCA1 fire when the animal locates in a particular subregion of its environment (O’Keefe and Dostrovsky, 1971). These “place cells” encode complex associations available in different locations (Best et al., 2001). In mice, damages to the dCA1 affect spatial cognition (Cheng and Ji, 2013). It is reported that disturbed rhythmic organization of place cell activity contributes to an unstable spatial representations and the related spatial memory deficits (Mably et al., 2017).

## BLA-Hippocampus Interactions

The studies discussed above strongly suggest that BLA and hippocampus are the two brain regions which can operate independently to exert their distinct functions in emotion and memory. However, some other studies suggest that BLA and hippocampus can also act synergistically. In anxiety-related behaviors, neurons in BLA (Wang et al., 2011) and hippocampus (Adhikari et al., 2010, 2011) fire actively, indicating their neural correlation. In contextual fear conditioning, inactivation of the BLA with muscimol, a GABAA receptor agonist, attenuates the consolidation of hippocampus-dependent context memory (Huff and Rudy, 2004; Huff et al., 2005). In addition, BLA manipulation alters genes expression (Packard et al., 1995) and synaptic plasticity of the hippocampus (Ikegaya et al., 1996; Akirav and Richter-Levin, 1999). Although these studies indicate that BLA could modulate hippocampus-dependent behavior via their neural correlation, it should be noted that these effects are not necessarily indicative of a direct, monosynaptic BLA-hippocampus projection, because these pharmaceutical injection of BLA and *in vivo* electrophysiology recording may inevitably manipulate multiple circuits between BLA and hippocampus. The mPFC is one of such regions forming synapses with both BLA and hippocampus. It bidirectionally connects with amygdala (Ghashghaei and Barbas, 2002; Ghashghaei et al., 2007; Delli Pizzi et al., 2017a,b) and receives projections from hippocampus simultaneously (Verwer et al., 1997; Parent et al., 2010). Thus, dissection of monosynaptic BLA-hippocampus projection is urgently needed to elucidate how BLA and hippocampus interact directly to account for emotion-regulated memories.

## Identification of Structural BLA–CA1 Circuitry

**Figure 2:**
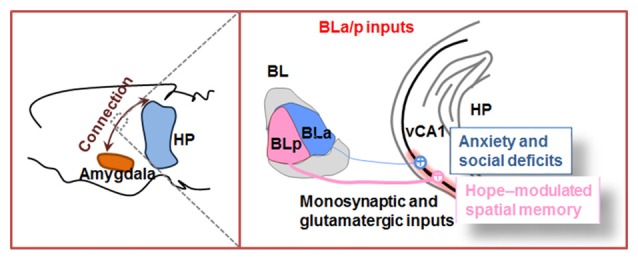
Outline of amygdala to hippocampal CA1 inputs. The basolateral amygdala nucleus (BL) is divided into anterior part (BLa) and posterior part (BLp). Both BLa and BLp project directly to ventral hippocampal CA1 (vCA1) and establish monosynaptic and glutamatergic circuits. In physiological condition, BLp–vCA1 projection is more intensive than BLa–vCA1 projection. Activation of BLa–vCA1 inputs induces anxiety and social deficits; while activation of BLp–vCA1 inputs mediates HOPE-facilitated spatial memory.

Projection tracers provide a possibility to describe the synaptic connections among brain regions. Since CA1 is the output node of hippocampus, here, we take BLA–CA1 inputs as an example to introduce their direct anatomical connection and their unique functions on emotion-associated memories (Figure 2).

### Outline of BLA–vCA1 Circuit by Traditional Tracers

Traditional tracers had been widely employed to outline long-range projections between brain regions (Katz et al., 1984; Köbbert et al., 2000; Swanson, 2000; Vercelli et al., 2000). To visualize BLA projections, anterograde tracer phaseolus vulgaris-leucoagglutinin was injected into various divisions. Pikkarainen et al. (1999) found that BL is the most prominent divisions of BLA that innervate the stratum radiatum and stratum oriens of hippocampal CA1 and CA3. The BM projects to the stratum lacunosum-moleculare of CA1. Thus, anatomical projection from BL to CA1 identified by traditional tracing method indicates that BL may be a key subregion to modulate different stages of information processing within the hippocampal formation.

### Identification of BL–CA1 Circuit by Advanced Anterograde and Retrograde Monosynaptic Tracing

The conventional anterograde tracers can reveal axonal projections of upstreams in brain connections, but they cannot identify the features of the neural circuitry. Virus-delivered tracking system commendably overcomes this weakness and well maps the cell type-specific connections. After injecting the anterograde tracker (AAV5–CaMKIIa–hChR2–mCherry) into BL, Felix-Ortiz et al. (2013) and Yang et al. (2016) detected robust mCherry signals in ventral but not dorsal region of hippocampal CA1, suggesting excitatory neural projections from BL to vCA1.

Virus-delivered tracking system is adept at mapping the cell type-specific connection, but it may create ambiguity about whether the cells are directly or indirectly connected. To improve this, Yang et al. (2016) developed Cre-dependent helper virus to precisely control initial rabies virus infection in vCA1 and subsequent retrograde monosynaptic spreading. By using this advanced retrograde monosynaptic tracing, they confirmed BL–vCA1 monosynaptic connection. Further, they discovered that posterior part (BLp)–vCA1 connection is more prominent than the anterior part (BLa)–vCA1 connection in physiologycal condition, which offers a precise supplement for BLA, specially BL subregion outputs (Yang et al., 2016).

## Optogenetic Insights on Functional BL–vCA1 Circuitry

Optogenetics successfully combines optics with genetics to allow a high level of temporal and spatial control of specific neuronal circuits (Tye and Deisseroth, 2012). It integrates light-sensitive proteins, such as channelrhodopsin, halorhodopsin and archaerhodopsin, into cell membranes, and finally results in neural activation or inhibition via photostimulation-induced depolarization or hyperpolarization of neuronal membranes where opsins are expressed. By combining optogenetics with *ex vivo* brain slice recording, Felix-Ortiz et al. (2013) identified the excitatory monosynaptic connection of BLa–vCA1 input. Then, the functional connection of BLp–vCA1 input was proved by Yang et al. (2016) *in vivo* extracellular recordings combined with optogenetic stimulation. Yang et al. (2016) first injected AAV5–CaMKIIα–hChR2–mCherry into BLp, and then photostimulated BLp fibers terminals in vCA1. They found that the average firing rate of vCA1 pyramidal neurons is identical to the photostimulation frequency. Their responding latency indicates monosynaptic connection of BL–vCA1 input (Yang et al., 2016).

### BLa–vCA1 Inputs Mediates Anxiety and Social Deficits

Numerous lines of evidence support that both BL and ventral hippocampus are responsible for the expression of anxiety-related behaviors (see “Basolateral Nucleus” and “Anatomy and Physiology of Hippocampus Formation” sections). However, the contribution of the monosynaptic connection between them is poorly understood. To identify the role of BLa–vCA1 inputs in anxiety behavior, light-sensitive opsins, i.e., ChR2 and NpHR, were expressed in BLa excitatory neurons and an optical fiber was implanted into vCA1 for the subsequent illumination on BLa-projected terminals. In line with hypotheses that amygdala hyperactivity underlies anxiety (Anagnostaras et al., 1999; Drevets, 2003; Carter and Krug, 2009), *in vivo* photoactivation of BLa–vCA1 synapses significantly increases anxiety-related behaviors, while photoinhibition produces robust anxiolytic effects. Combining optogenetic approaches with *in vivo* pharmacological manipulations, light-elicited anxiogenic effects were completely prevented by intra-vCA1 injection of glutamate antagonism, demonstrating that glutamatergic excitatory projections from the BLa to the vCA1 are sufficient to mediate anxiety. Thus, opposite to CeL, vCA1 is an important anxiogenic downstream target of BL. Monosynaptic BLa–vCA1 projection could control anxiety-related behaviors in a bidirectional and reversible manner.

Along with the role in anxiety, both BL and ventral hippocampus are important in social behaviors (see “Basolateral Nucleus” and “Anatomy and Physiology of Hippocampus Formation” sections). Using the approach described above to target BLa–vCA1 inputs, Felix-Ortiz et al. (2013) found that photoinhibition increases, while photoactivation decreases social behaviors as shown in the resident-juvenile intruder procedure. Again, intra-vCA1 injection of glutamate receptor antagonism significantly abolished the social deficits induced by photoactivation (Felix-Ortiz and Tye, 2014). Therefore, vCA1 is a candidate target that forms circuitry with BL to control social behaviors in a bidirectional, immediate, yet reversible manner.

Taken together, these two optogenetic studies reveal that BLa can directly activate vCA1 controlling both anxiety and social behaviors. Since anxiety is often co-expressed with social dysfunction, these findings provide evidence that BLa–vCA1 inputs may be a key mechanism at neural circuit linking the co-morbidity of anxiety disorders and social deficits.

### BLp–vCA1 Inputs Control Emotion-Modulated Spatial Memory

In parallel to the key role in negative emotions, BL also participates in positive emotions. Work in rodents has shown that negative and positive emotion neurons are spatially segregated into the BLa and BLp (Kim et al., 2016). By monosynaptic tracing, Yang et al. (2016) found that the BLp–vCA1 connections were much stronger than that of BLa–vCA1. By exposing rodents to inescapable footshocks with avoidance trainings, Yang et al. (2016) established a novel animal model termed “HOPE” (i.e., the learnt hopeful, LHF) or with positive motivation in face of pressure. They found that the HOPE animals show potentiated spatial memory with up-scaling of BLp–vCA1 excitatory inputs, whereas the learnt helpless (LHL) animals show impaired spatial memory with a suppressed BLp–vCA1 connection. Manipulating BLp–vCA1 inputs in a same manner as described in BLa–vCA1 connection, they found that photoinhibition of BLp–vCA1 inputs abolished the facilitative effects of LHF and impairs synaptic plasticity. By contrast, photoactivation of BLp–vCA1 inputs rescued the LHL-induced memory impairments and mimics the positive effects of LHF. Stimulation of BLp–vCA1 could upregulate CREB and intrasynaptic AMPA receptors with an enhanced synaptic transmission in CA1. Thus, unlike the role of BLa–vCA1 inputs in negative emotion, BLp–vCA1 connections gate positive emotion-facilitated spatial memory. Although emotion-enhanced memory likely involve a distributed neural network across multiple brain regions, BLp–vCA1 glutamatergic inputs are sufficient to mediate HOPE-potentiated spatial memory. Given that most patients with senile dementia displayed emotional disorder and spatial memory impairment, this study provides new insight into the pathogenesis of these emotion-associated diseases, and the discovery of BLp–vCA1 circuit provides a potential target for the treatment of deep brain stimulation (DBS; Yang et al., 2016).

## Future Directions

The amygdala and hippocampal complex govern two independent memory systems that interact when emotion meets memory. We are just beginning to understand the subtleties of these interactions and there are still some unanswered key questions. In further development of the neuroanatomical circuits discussed here, three directions should be emphasized.

First, it is important to better define BLA functional microcircuitry by which information is highly integrated before output to the innervated downstream targets, including hippocampus. Glutamatergic and GABAergic sets are the two non-overlapping populationsin BLA (Sah et al., 2003). Spiny glutamatergic neurons account for 80% of BLA, while sparsely spiny GABAergic interneurons for 20%. Among five types of GABAergic interneurons (McDonald and Betette, 2001; McDonald and Mascagni, 2001, 2002; Mascagni and McDonald, 2003, 2007; Spampanato et al., 2011), parvalbumin (PV+) or somatostatin (SOM+) interneurons are the two main classes within BLA. They regulate principal cells in distinct ways (Smith et al., 2000; Muller et al., 2005, 2006, 2007). For instance, PV+ interneurons preferentially target the perisomatic region of their target cells, such as principal cells and SOM+ interneurons, by which control activity and spike output of these targeted neurons (McDonald and Betette, 2001; Somogyi and Klausberger, 2005; Muller et al., 2006). In contrast, SOM^+^ interneurons preferentially form synapses at the distal dendrites of principal cells (Muller et al., 2007), by which efficiently control the impact of inputs to their target cells (Gentet et al., 2012; Chiu et al., 2013). More investigation will be required to determine the nature and difference of valence-associated neurons (i.e., glutamatergic and/or GABAergic neurons) within the BLA microcircuitry, to better understand how they are specifically activated according to the emotion valences and how they can work synergistically.

Second, it is also important to define the functional microcircuitry of hippocampus which is directly or indirectly innervated by BLA. By *ex vivo* brain slice recording and *in vivo* recording, Felix-Ortiz et al. (2013) and Yang et al. (2016) have identified the excitatory, monosynaptic glutamatergic inputs from BLa/p to vCA1 pyramidal neurons. However, whether BLA neurons, including glutamatergic and GABAergic neurons, directly or indirectly innervate vCA1 glutamatergic and GABAergic neurons is largely unknown. Those circuits’ functions and interaction also need further investigation. In addition, identification of the projection differences between posterior and anterior part, magnocellular and parvicellular subdivisions of BLA to vCA1 would provide more details to outline BLA-hippocampus circuits.

Third, it is critical to better translate the experimental results from animals to humans. In clinic, there are quite numbers of psychiatric patients and those with cognition deficits who are partially or totally resistant to conventional medicine therapies. DBS brings hope for these patients. It should be realized that therapeutic effect of DBS depends on the appropriate selection of targets in neural circuits mediating diseases. However, lacking spatial specificity is the big limitation of DBS treatment (Benabid, 2015). In the electrical stimulated filed, whether and how far the DBS-induced electrical pulses spread is greatly dependent on the tissue conductivity. So, in brain neural circuitry, the distinct conductivity leads currents to spread unevenly in all directions (Benabid, 2015). Furthermore, properties of neuronal composition can differentially affect excitability, thus DBS can modulate distinct functions in tissues that possess unique neuronal compositions (Li et al., 2012). In short, it seems hard to predict accurately what effects DBS will exert finally (Benabid, 2015). In view of the above evidence, tailoring DBS by absorbing the merits of optogenetics will be urgently desired to translate animal studies to clinics, by which reliable and no side-effects treatments for patients could be expected to achieve.

## Author Contributions

All authors listed, have made substantial, direct and intellectual contribution to the work, and approved it for publication.

## Conflict of Interest Statement

The authors declare that the research was conducted in the absence of any commercial or financial relationships that could be construed as a potential conflict of interest.
